# Transcriptome of human neuroblastoma SH-SY5Y cells in response to 2B protein of enterovirus-A71

**DOI:** 10.1038/s41598-022-05904-6

**Published:** 2022-02-02

**Authors:** Kittisak Suanpan, Potjanee Srimanote, Pongsri Tongtawe, Onruedee Khantisitthiporn, Oratai Supasorn, Patthaya Rattanakomol, Jeeraphong Thanongsaksrikul

**Affiliations:** 1 Graduate Program in Biomedical Sciences, Faculty of Allied Health Sciences, Thammasat University, Pathum Thani, 12120 Thailand; 2 Thammasat University Research Unit in Molecular Pathogenesis and Immunology of Infectious Diseases, Thammasat University, Pathum Thani, 12120 Thailand; 3 Department of Medical Technology, Faculty of Allied Health Sciences, Thammasat University, Pathum Thani, 12120 Thailand

**Keywords:** Immunology, Microbiology, Molecular biology

## Abstract

Infection with enterovirus-A71 (EV-A71) can cause hand-foot-mouth disease associated with fatal neurological complications. The host response to EV-A71 has not yet been fully elucidated, thus, hampering the development of a precise therapeutic approach. A nonstructural 2B protein of EV-A71 has been reported to involve with calcium dysregulation and apoptosis induction in human neuroblastoma SH-SY5Y cells. However, the molecular mechanism has not been delineated. To address this, comprehensive study of the gene expression from SH-SY5Y cells transfected with EV-A71 2B was carried out by RNA sequencing and transcriptomic analysis. It was found that the signature of the upregulated genes of SH-SY5Y cells expressing EV-A71 2B involved the Ca^2+^-related signaling pathways participating gene expression, inflammatory response, apoptosis, and long-term potentiation of the neuron. Protein–protein interaction network analysis revealed that the products encoded by *CCL2*, *RELB*, *BIRC3*, and *TNFRSF9* were the most significant hub proteins in the network. It indicated that EV-A71 2B protein might play a role in immunopathogenesis of the central nervous system (CNS) which probably associated with the non-canonical NF-κB pathway. The data suggest that transcriptomic profiling can provide novel information source for studying the neuropathogenesis of EV-A71 infection leading to development of an effective therapeutic measure for CNS complications.

## Introduction

Enterovirus-A71 (EV-A71) is a small non-enveloped, positive-sense, single-stranded RNA virus (+ ssRNA)^1^. It has been classified in *Enterovirus* A genus, *Picornaviridae* family. EV-A71 is one of the causative agents of hand-foot-mouth disease associated with neurological complications including aseptic meningitis, brainstem encephalitis, flaccid paralysis, and neurogenic cardiopulmonary failure^2,3^. Pathological changes found in the EV-A71-infected cells have been associated with virus-host interactions leading to dysregulated homeostasis of various cellular responses such as cellular metabolism, immune responses, inflammation, and cell death^4,5^. The degeneration of the infected neurons has been postulated to be involved in the neuropathogenesis of the EV-A71 infection. However, the exact molecular mechanisms or pathways that involve the neuropathogenesis have not been elucidated resulting in dampening the development of therapeutic and preventive strategies for fatal complications of the EV-A71 infection.

Properly controlled homeostasis of calcium (Ca^2+^) and tightly regulated calcium-dependent signal transduction pathways have been implicated in normal brain physiology and neuronal cell integrity and survival^6,7^. Emerging knowledge indicates that dysregulated calcium homeostasis in neuronal cells could trigger complex and diverse signal transduction pathways that lead to neurodegenerative disorders such as Alzheimer’s and Parkinson’s diseases^8,9^. In the case of EV-A71 infection, our previous publication has reported that the nonstructural 2B protein of the EV-A71 could increase cytosolic Ca^2+^ at 6 h post-transfection and induce apoptosis in the transfected human neuroblastoma SH-SY5Y cells. The upregulation of caspase-9 (*CASP9*) and (*CASP12*) transcription was initially detected at 3- and 24 h post-transfection, respectively, in the transfected SH-SY5Y cells^10^. It indicated the involvement of ER-mitochondrial interaction triggered by mobilization of Ca^2+^ between ER and mitochondria leading to disturbance of normal functions of the organelles and activation of caspase-dependent intrinsic apoptosis pathways in the EV-A71 2B transfected SH-SY5Y cells. Elucidation of gene expression profiles of SH-SY5Y cells expressing EV-A71 2B might allow us to gain the comprehensive knowledge of neuropathogenesis mediated by EV-A71 infection. Nonetheless, the network of signal transduction pathways in the neuronal cells in response to aberrant increase of the cytosolic Ca^2+^ mediated by EV-A71 2B protein has not been elucidated.

In this study, transcriptomes of human neuroblastoma SH-SY5Y cells expressing the recombinant EV-A71 2B protein fused with FLAG tag and mCherry, namely 2BmCherry, were analyzed and compared with transcriptomes of the control groups including SH-SY5Y cells expressing mCherry and normal cells. The comparative transcriptomic analysis was used to profile the differentially expressed genes of the potential signal transduction pathways triggered by the EV-A71 2B protein. The gained knowledge will provide not only new insight into the underlying mechanisms of the virus-host interactions leading to neuropathogenesis of the EV-A71 infection but also the target candidates for therapeutics.

## Results

### Global view of the host transcriptional responses in human SH-SY5Y cells transfected with EV-A71 non-structural 2B protein

**Figure 1:**
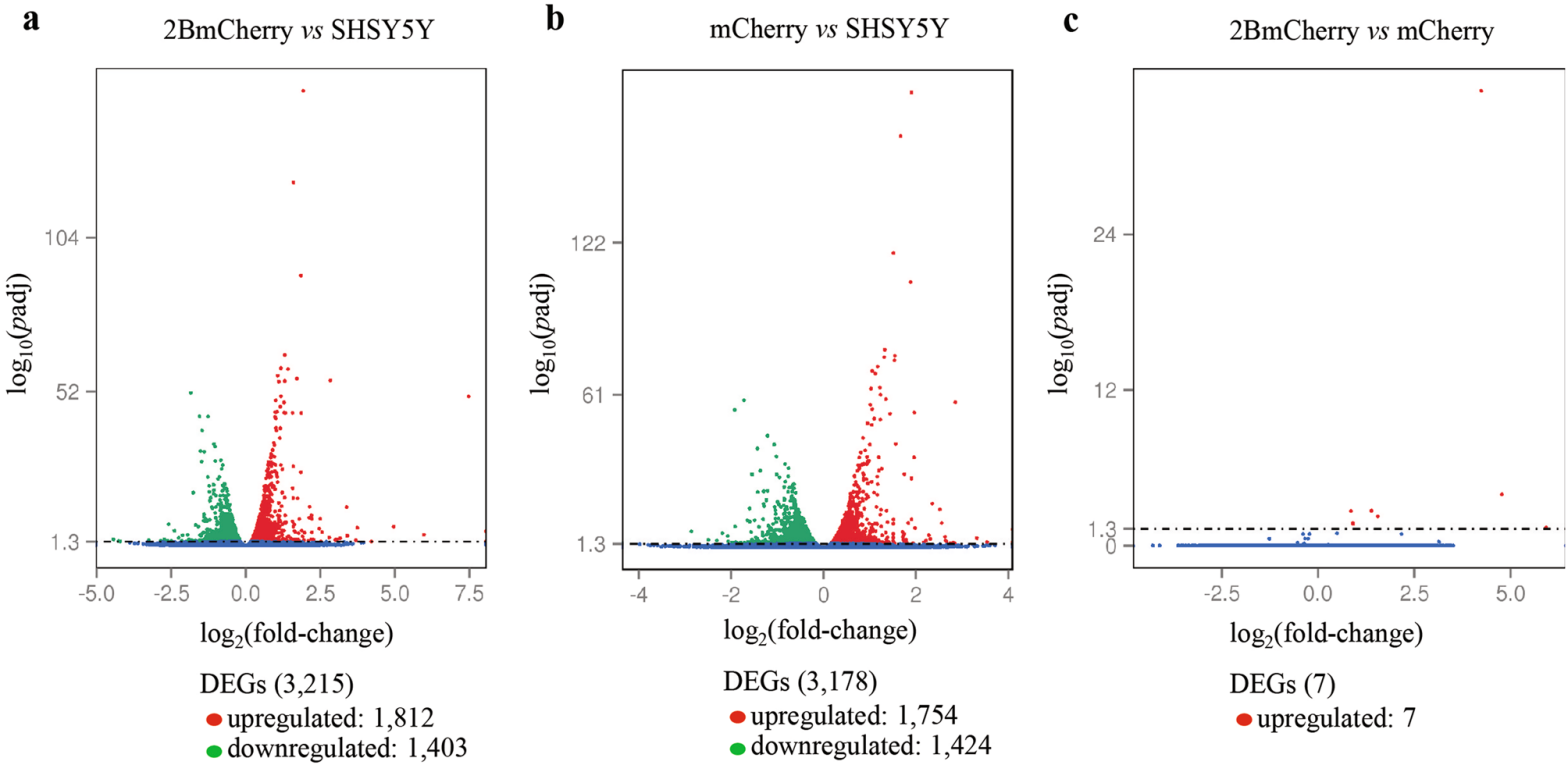
Volcano plots of the differentially expressed genes (DEGs) in each comparison group including 2BmCherry versus SHSY5Y transcriptome (**a**) mCherry versus SHSY5Y transcriptome (**b**), and 2BmCherry versus mCherry transcriptome (**c**). The *x*-axis represents the magnitude of statistical significance. The *y*-axis indicates the magnitude of change. The dash line indicates a threshold of significant magnitude at -log_10_(*p-*adj) = 1.3 or *p*-adj < 0.05.

To characterize the response of the human neuronal cells to the EV-A71 2B protein, the transcriptomic data from the SH-SY5Y cells transfected with the EV-A71 2B protein fused with FLAG tag and mCherry, designated as 2BmCherry, were analyzed and compared with the data of cells expressing mCherry, designated as mCherry and served as the background control, and the normal cells, designated as SHSY5Y. Bases on *p-*adj < 0.05, it was found that 3851 differentially expressed genes (DEGs) among the three transcriptomes were identified by pairwise comparison analysis (2BmCherry versus SHSY5Y, mCherry versus SHSY5Y, and 2BmCherry versus mCherry). The overview of transcriptional changes was shown by volcano plots that were drawn between the magnitude of statistically significant differences and the magnitude of changes (Fig. 1). A total of 3251 DEGs were discovered in comparison between 2BmCherry versus SHSY5Y transcriptomes including 1812 upregulated- and 1403 downregulated genes (Fig. 1a). In comparison between the mCherry versus SHSY5Y transcriptomes, 3178 DEGs were identified including 1754 upregulated- and 1424 downregulated genes (Fig. 1b). There were 7 upregulated DEGs identified from the comparison between the 2BmCherry versus mCherry transcriptomes (Fig. 1c). Data of read quality and read mapping analysis of the three transcriptomes were provided in Supplementary Tables 1 and 2.

### Patterns of the putative host cellular responses in human neuroblastoma SH-SY5Y cells transfected with EV-A71 non-structural 2B protein

**Figure 2:**
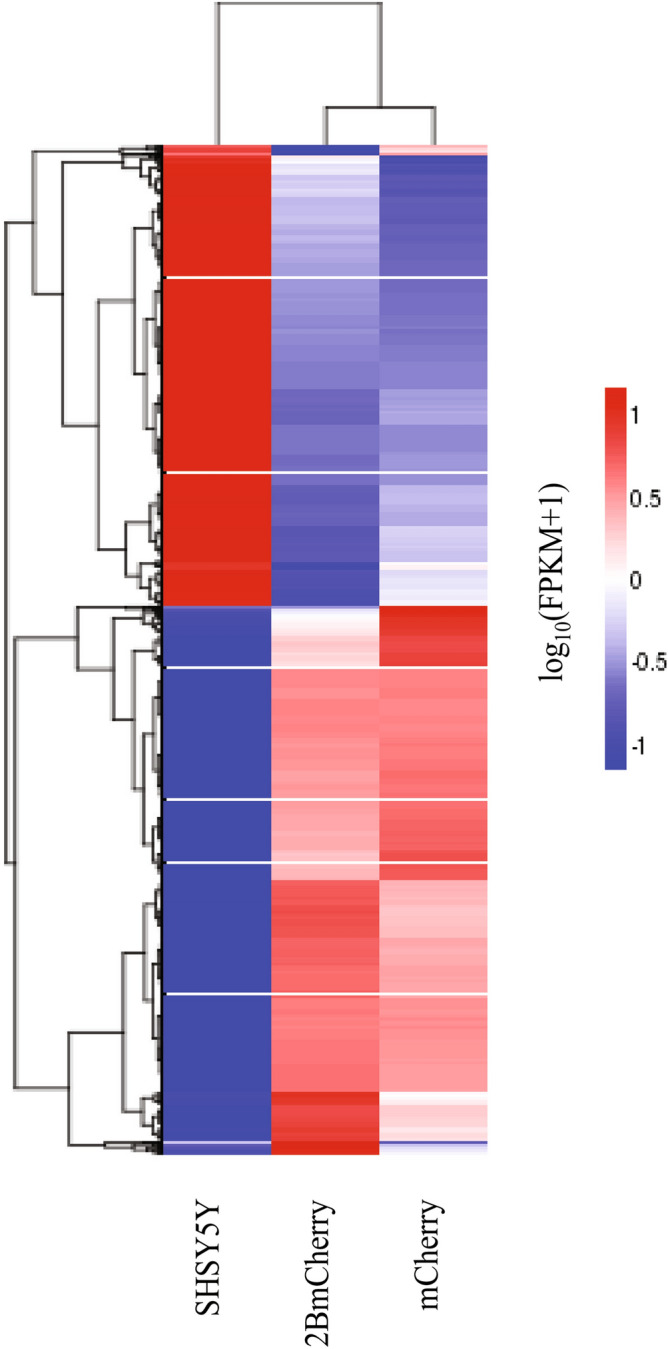
Unsupervised hierarchical heatmap clustering with double-dendrogram of the patterns of significant differences of DEGs among the three transcriptomes. DEGs are clustered in roll of each transcriptome displayed on the column. The gradient colors indicate the magnitude of changes in the gene expression levels, red color for upregulation and blue color for downregulation. Dendrogram on the top indicates a cluster of the genes with similar expression levels. Dendrogram on the left site indicates a similarity of the expression pattern across the transcriptome.

**Figure 3:**
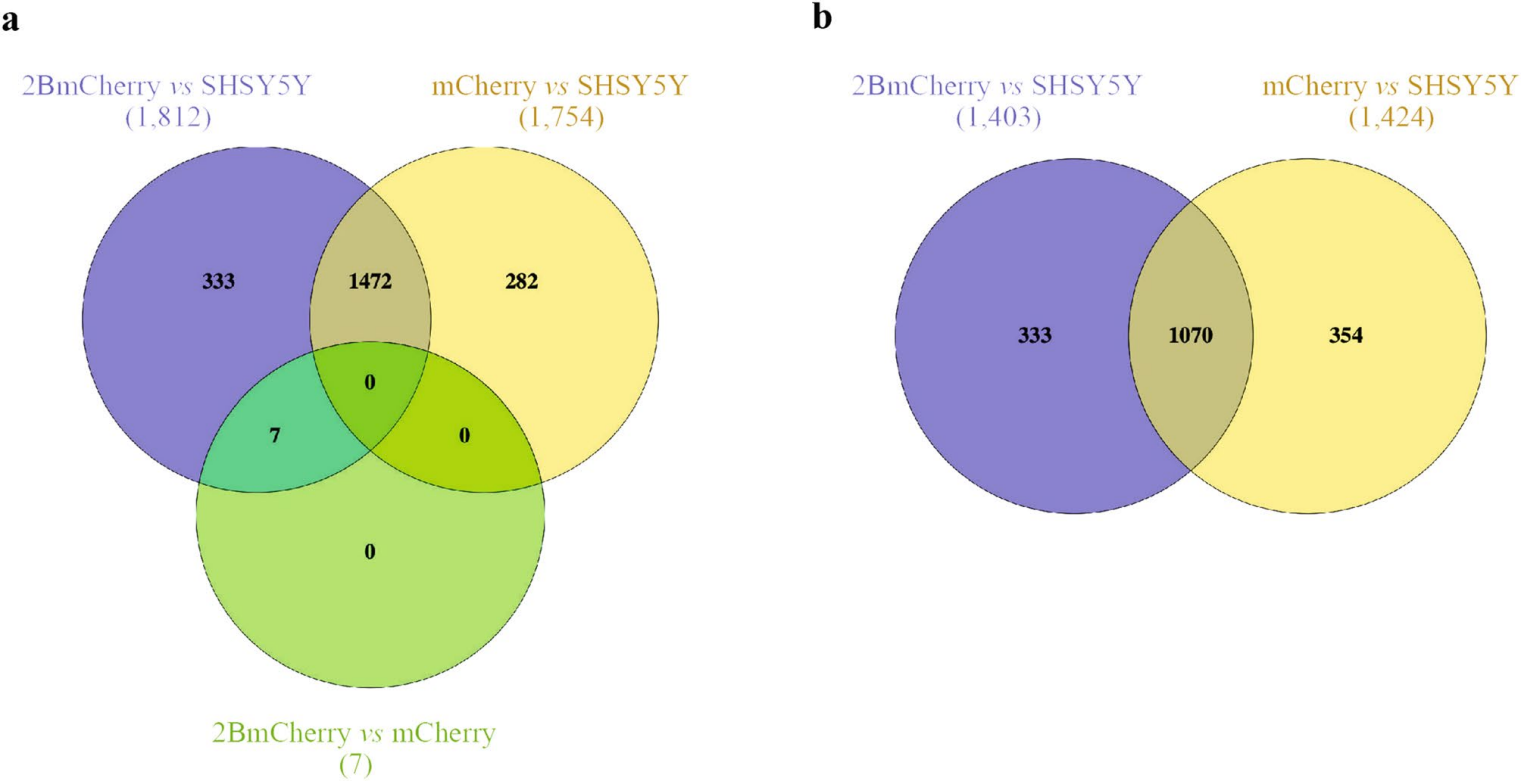
Venn diagrams of upregulated (**a**) and downregulated (**b**) DEGs among the three comparison groups (2BmCherry versus SHSY5Y, mCherry versus SHSY5Y, and 2BmCherry versus mCherry). The numbers in the overlap areas indicate the common DEGs found in the compared transcriptomes. The numbers in the non-overlap areas indicate the unique DEGs of the respective transcriptomes.

To reveal the patterns of DEGs expression among the three transcriptomes, unsupervised hierarchical heatmap clustering (h-cluster) analysis with the two-way dendrogram was performed. It was found that 3851 DEGs were clustered and divided into two main distinct clusters of 2094 upregulated DEGs and 1757 downregulated DEGs (Fig. 2). The heatmap and the dendrogram showed that the patterns of the 2BmCherry transcriptome are closely related to the mCherry transcriptome. Although the DEG patterns were most likely similar, however, the expression level value could be distinguished between them clearly. H-subcluster analysis classified 3851 DEGs into 28 subclusters, some of which showed the different patterns between DEGs from 2BmCherry and mCherry (Supplementary Fig. 1). On the other hand, they were different from the SHSY5Y transcriptome. Moreover, the set of DEGs in each pairwise comparison transcriptome illustrated by the Venn diagram showed both a set of genes which were signature in 2BmCherry transcriptome and common with the other transcriptomes (Fig. 3). Among the 2094 upregulated DEGs (Fig. 3a), 340 upregulated DEGs were uniquely expressed in the 2BmCherry transcriptome, of which 333 and 7 DEGs were identified in comparison with the SHSY5Y and the mCherry transcriptomes, respectively. The fold-changes of the expression level of the 333 upregulated DEGs showed a statistically significant difference (*p*-adj < 0.05) in the comparison between 2BmCherry and SHSY5Y transcriptomes but were not different in the mCherry versus SHSY5Y comparison (Supplementary Table 3). The seven DEGs of 2BmCherry transcriptome that showed overexpression in comparison to mCherry included *CCL2*, *RELB*, *IL32*, *PLAT*, *PTGES*, *PHLDA1*, and *TNFRSF9*. Among the 1757 downregulated DEGs (Fig. 3b), there were 333 DEGs that were uniquely found in the 2BmCherry transcriptome in comparison with the SHSY5Y transcriptome. It was suggested that the 340 upregulated and the 333 downregulated DEGs could be considered as the transcriptomic signature of the human neuronal cells in response to EV-A71 non-structural 2B protein. The dataset of the upregulated- and the downregulated DEGs were supplied as Supplementary Table 3.

### Validation of the expression of the selected unique DEGs of 2BmCherry transcriptome

To validate the expression of DEGs, the seven genes from 2BmCherry transcriptome that showed the unique overexpression in comparison with mCherry and SHSY5Y transcriptomes were selected and analyzed by qRT-PCR. These genes included *CCL2*, *RELB*, *IL32*, *PLAT*, *PTGES*, *PHLDA1* and *TNFRSF9*. The relative fold-changes of the genes in the human neuroblastoma SH-SY5Y cells transfected with recombinant plasmid expressing 2BmCherry, namely *pLVX-puro::FLAG-2B-mCherry*, were calculated by the relative Ct values to those of the cells transfected with recombinant plasmid expressing mCherry, namely *pLenti*::*mCherry*, normalized with the Ct value of the respective gene amplified from the normal cells by 2^−∆∆Ct^ method. It was found that the relative fold-changes of *CCL2*, *IL32*, *PHLDA1*, and *TNFRSF9* were highly up-expressed as determined by qRT-PCR. While *RELB* and *PTGES* showed the marginal high relative fold-changes. Unfortunately, amplification of *PLAT* was failed after several attempts by alternative specific primers even the optimized conditions had been performed. The relative fold-changes of the selected genes determined by qRT-PCR comparing to the data derived from RNA-sequencing analysis were shown in Table 1.

**Table 1:** The comparison of fold-changes in the expression of the selected 7 DEGs in SH-SY5Y cells transfected with 2BmCherry determined by RNA-sequencing analysis and qRT-PCR amplification.

Gene	Log_2_ (fold change)	
	RNA-sequencing analysis	qRT-PCR ± SD
*CCL2*	4.24	0.55 ± 0.37
*RELB*	1.39	− 0.16 ± 0.25
*IL32*	4.78	0.32 ± 0.48
*PLAT*	0.91	Undetermined
*PTGES*	0.85	0.02 ± 0.27
*PHLDA1*	1.55	0.31 ± 0.42
*TNFRSF9*	5.92	0.51 ± 0.33

### Functional characteristics of the unique DEGs of the 2BmCherry transcriptome

To classify the biological meaning and the molecular network in human neuronal SH-SY5Y cells in response to EV-A71 non-structural 2B protein, the DEGs that were unique to the 2BmCherry transcriptome were analyzed by Gene Ontology (GO) enrichment analysis and Kyoto encyclopedia of genes and genomes (KEGG) pathway enrichment analysis.

**Figure 4:**
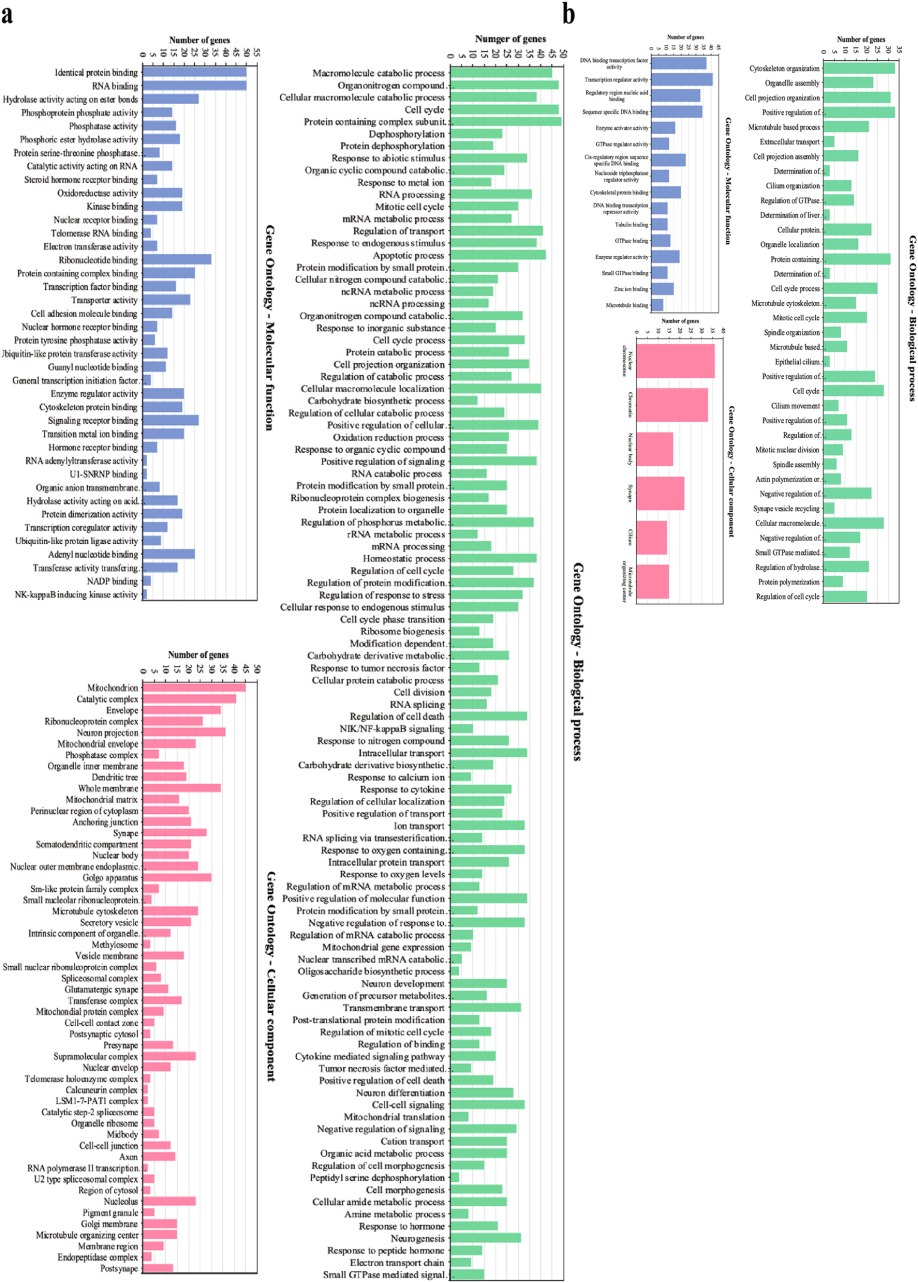
The bar charts of the significant GO categories enriched by the upregulated (**a**) and the downregulated (**b**) DEGs in the 2BmCherry transcriptome. The *x*-axis represents the name of a certain GO term. The *y*-axis refers to the number of the genes enriched in the term.

GO analysis among the 673 unique DEGs in 2BmCherry transcriptome revealed that 594 DEGs including 328 upregulated- and 266 downregulated DEGs could be classified into the significant GO categories (Fig. 4). It was shown that the uniquely upregulated DEGs were enriched in GO:BP (biological process) involved in responses to calcium ion, dephosphorylation, NIK/NF-κB (non-canonical NF-κB pathway), signal transduction, response to cytokine, and tumor necrosis factor which involved with NFKB1 (in canonical NF-κB pathway), gene expression, cellular metabolism, cell cycle, cell signaling pathways, cellular homeostasis, electron transport chain and energy consumption, neuron differentiation and development, intracellular transport, and apoptosis (Fig. 4a). In GO:MF (molecular function), the uniquely upregulated DEGs involved with binding and catalytic activity on both protein and nucleic acid, phosphorylation and phosphatase activity, ubiquitin-like protein ligase and transferase activity, oxidoreductase activity, and NF-κB inducing kinase activity. In terms of GO:CC (cellular component), the uniquely upregulated DEGs were localized at the whole plasma membrane of a neuron, both pre- and post-synapse, and cellular membranes such as mitochondria, ER, Golgi apparatus, and vesicles as well as protein complexes such as spliceosomal complex, telomerase holoenzyme complex, microtubule organizing center, mitochondrial protein complex, and calcineurin complex. The uniquely downregulated DEGs were enriched in GO:BP involved cytoskeleton organization, structures and functions of neurons, and regulation of cell cycle (Fig. 4b). The uniquely downregulated DEGs were enriched in GO:MF associated with regulation of transcription and binding activity such as cytoskeleton binding and GTPase binding. In terms of GO:CC, the uniquely downregulated DEGs localized at nucleus, microtubule organizing center, and membrane of the neuron. These results indicated that the Ca^2+^-related signaling pathways, NIK/NF-κB (non-canonical NF-κB) pathways, mitochondria dysregulation, cellular processes, and apoptosis in human neuronal SH-SY5Y cells were triggered by the EV-A71 non-structural 2B protein which could affect metabolism, gene expression, cell survival, and normal functions of the neurons. For KEGG analysis, it was found that among the 673 unique DEGs in 2BmCherry transcriptome, only 328 upregulated DEGs could be annotated to the KEGG pathways. The most significant pathways at FDR *q*-value < 0.05 were listed in Table 2. It was shown that the uniquely upregulated DEGs in the 2BmCherry transcriptome were associated with cell survival and death, including apoptosis, oocyte meiosis, and small cell lung cancer. Certain pathways were related to the cellular biological processes including spliceosome, neuron long-term potentiation, and RNA degradation. Moreover, the pathways involved in the signal transduction revealed including the Wnt-signaling pathway, NOD-like receptor signaling pathway, MAPK-signaling pathway, and T-cell receptor signaling pathway. Further, these signaling pathways included apoptosis, cell cycle (oocyte meiosis), Wnt, MAPK, immune response, and long-term potentiation (adaptation of neuronal synaptic activity) and seemed to be associated with Ca^2+^ as the *PPP3CA* encoding for calcineurin was upregulated in the pathways. Cytokine production in the human SH-SY5Y cells probably was induced by the EV-A71 2B protein since *NFKB1* and *CCL2* were up-expressed in the pathways of T-cell receptor and NOD-like receptor signal transduction. Furthermore, NIK/NF-κB, or non-canonical NF-κB, pathway might play an important role in human neuronal SH-SY5Y cells in response to the EV-A71 non-structural 2B protein as *RELB*, *TRAF2*, or *BIRC3* were upregulated. This could be implied that the Ca^2+^-related signaling pathways involved with gene expression, immune response, apoptosis, and long-term potentiation (adaptation of synaptic activity) of the human neuronal SH-SY5Y cells might be activated by EV-A71 non-structural 2B protein and probably associated with the non-canonical NF-κB pathway. The datasets of GOs and KEGGs of the unique upregulated– and downregulated DEGs of the 2BmCherry transcriptome were supplied as Supplementary Tables 4 and 5, respectively.

**Table 2:** Lists of significant unique KEGG pathways of upregulated GO terms in 2BmCherry transcriptome^11^.

KEGG ID	KEGG pathway	Gene in overlap (k)	Gene in gene set (K)	k/K	*p*-value	FDR *q*-value	Gene
hsa04210	Apoptosis	8	87	0.09	6.17E−07	1.15E−04	*CHP1*,* PPP3CA*,* PPP3R1*,* NFKB1*,* BIRC3*,* TRAF2*,* IRAK1*,* EXOG*
hsa03040	Spliceosome	8	127	0.06	1.07E−05	9.92E−04	*LSM3*,* SNRPB*,* USP39*,* HNRNPC*,* NCBP1*,* SNRPC*,* PHF5A*,* SNRNP40*
hsa04114	Oocyte meiosis	7	113	0.06	4.25E−05	2.63E−03	*CHP1*,* PPP3CA*,* PPP3R1*,* PPP2CB*,* PPP2R1B*,* PPP1CA*,* YWHAH*
hsa05222	Small cell lung cancer	5	84	0.06	6.49E−04	3.02E−02	*NFKB1*,* BIRC3*,* TRAF2*,* CDK4*,* SKP2*
hsa03018	RNA degradation	4	59	0.07	1.41E−03	3.99E−02	*LSM3*,* EXOSC7*,* LSM1*,* EXOSC4*
hsa04310	Wnt-signaling pathway	6	151	0.04	1.59E−03	3.99E−02	*CHP1*,* PPP3CA*,* PPP3R1*,* PPP2CB*,* PPP2R1B*,* FZD9*
hsa04621	NOD-like receptor pathway	4	62	0.06	1.69E−03	3.99E−02	*NFKB1*,* BIRC3*,* CCL2*,* SUGT1*
hsa04010	MAPK-signaling pathway	8	267	0.03	1.72E−03	3.99E−02	*CHP1*,* PPP3CA*,* PPP3R1*,* NFKB1*,* TRAF2*,* RRAS2*,* DUSP3*,* RELB*
hsa04660	T-cell receptor signaling pathway	5	108	0.05	2.00E−03	4.13E−02	*CHP1*,* PPP3CA*,* PPP3R1*,* NFKB1*,* CDK4*
hsa04720	Long-term potentiation	4	70	0.06	2.64E−03	4.92E−02	*CHP1*,* PPP3CA*,* PPP3R1*,* PPP1CA*

### Protein–protein interaction (PPI) network of the uniquely upregulated and downregulated DEGs in the 2BmCherry transcriptome

**Figure 5:**
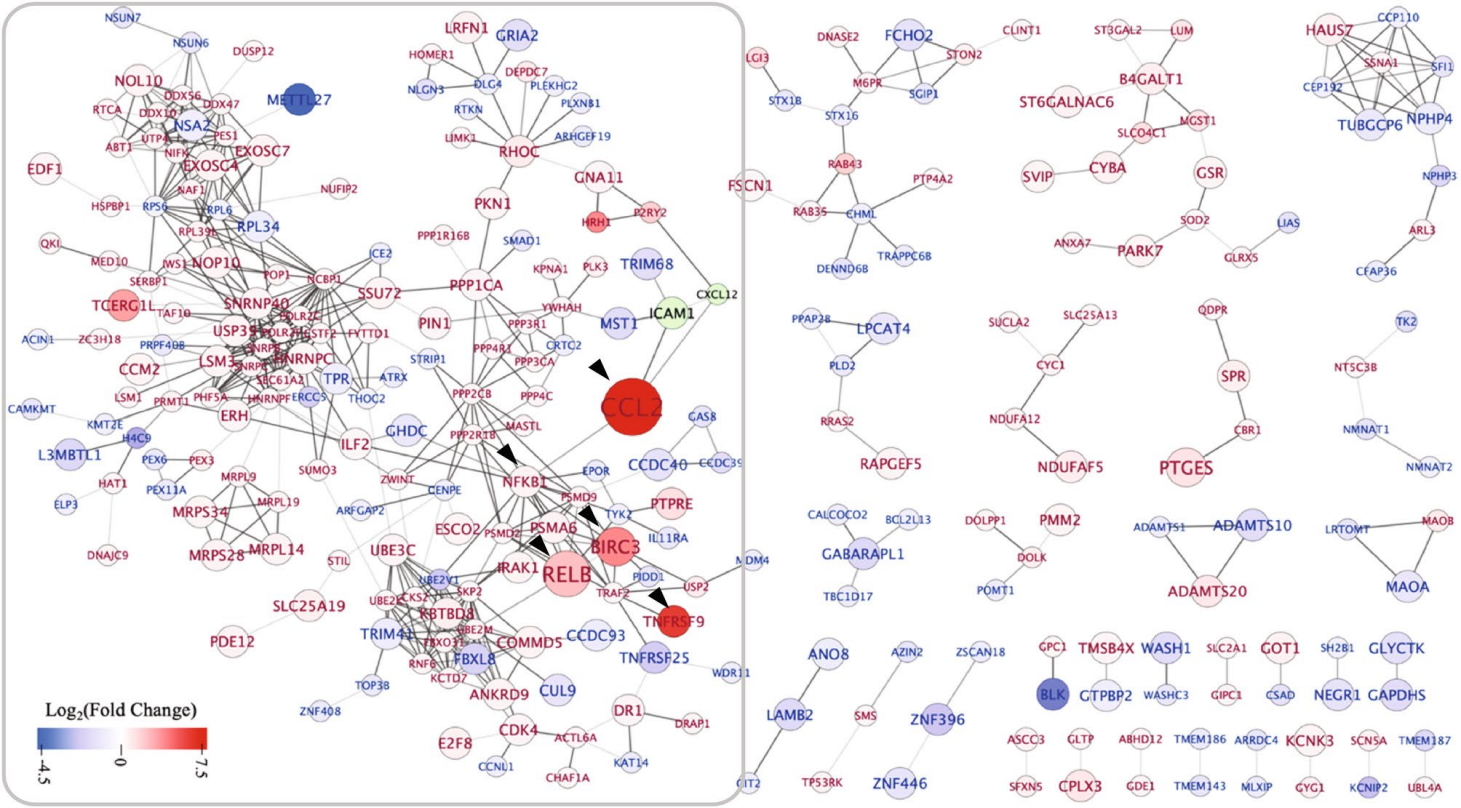
Diagram depicts the protein–protein interaction (PPI) network of the unique DEGs in the 2BmCherry transcriptome. The circles represent the protein nodes, the bigger size indicates the higher magnitude of statistical significance. The colors indicate the magnitude of changes, the log_2_(fold changes); red color indicates the upregulation, blue color indicates the downregulation, and green color indicates the undetermined fold change. The significant nodes are indicated by arrowheads. The light-to-dark grey colors of the connected lines indicate the increment of confidence of interaction (combined score 0.700–0.999). The nodes in the box show the biggest network.

**Figure 6:**
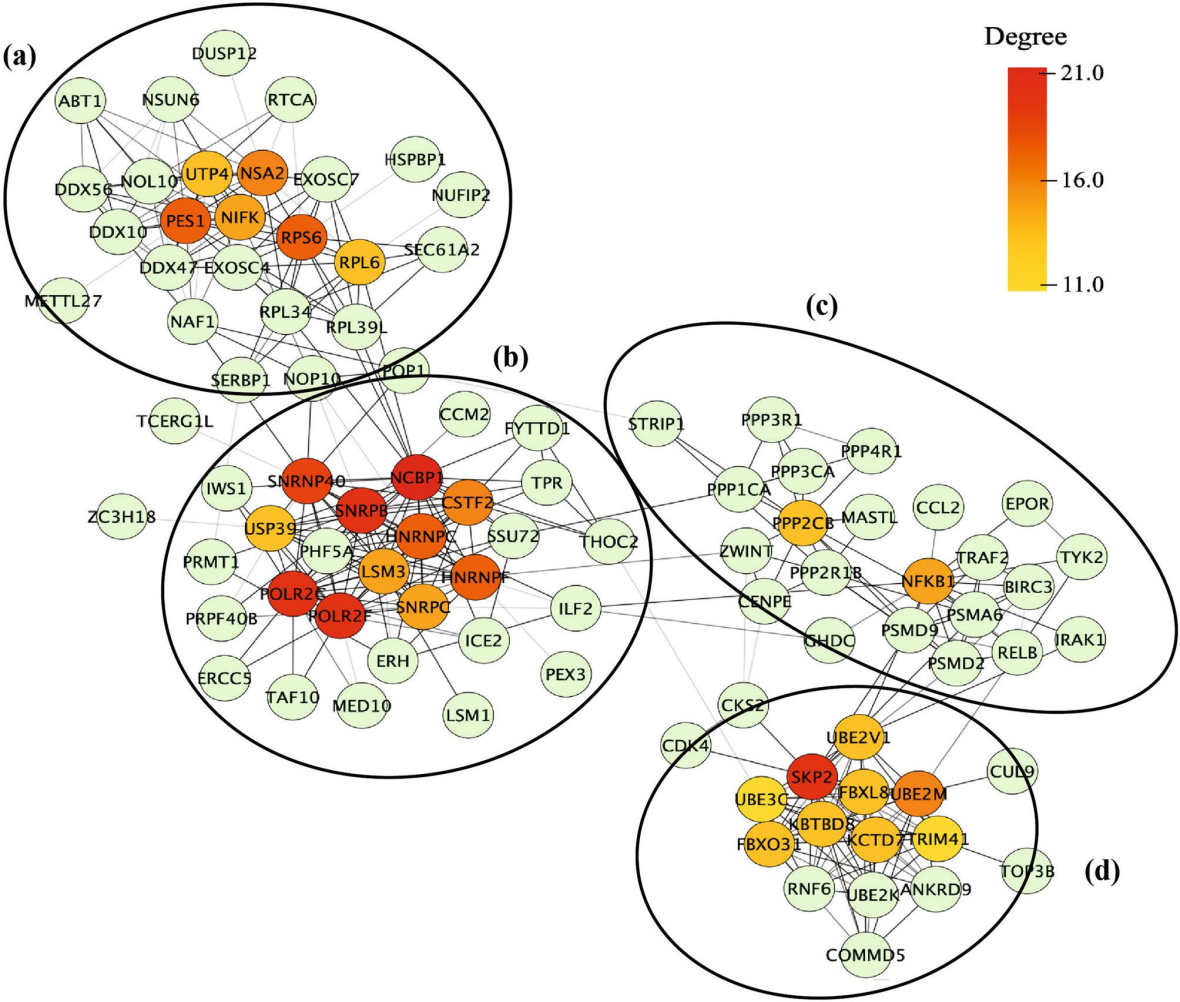
Diagram depicts the sub-network of the protein hubs and their first-neighboring interactors. The gradient color of yellow-to-red of the protein nodes indicates increment of the degree value for being the hub. The pale green nodes refer to the first-neighboring interactor in the network. The light-to-dark grey color of the connected lines indicates the confidence value of interaction (0.700–0.999). There are 4 clusters of nodes in which each of them is composed of DEGs involved in the regulation of gene expression (**a**), RNA processing (**b**), cell survival and cell death (**c**), and (**d**) protein degradation which indicated by the separate circles.

To evaluate the potential interaction of the DEGs, a PPI network analysis was conducted to predict the interaction between the unique DEGs of the 2BmCherry transcriptome. Among 673 unique DEGs identified in the 2BmCherry transcriptome, 594 DEGs including 327 upregulated- and 267 downregulated DEGs were annotated to the STRING protein database. PPI network of the DEGs was predicted and visualized (Fig. 5). It was found that there were 28 connected nodes with the highest degree as the protein hubs in the network. The most significant hub proteins included the products encoded by *CCL2*, *RELB*, *BIRC3*, and *TNFRSF9*. The *RELB*, *BIRC3*, and *TNFRSF9* were the genes in the non-canonical NF-κB, pathway. Sub-networks of the protein hubs with the first-neighboring node were analyzed. It was found that there were 4 clusters of the nodes in which their functions were related to the regulation of gene expression, RNA processing, cell survival and death, and protein degradation (Fig. 6).

## Discussion

Enterovirus-A71 (EV-A71) can infect the central nervous system (CNS) resulting in inflammation of brain parenchyma, brain stem, and spinal cord leading to neurodegeneration^12,13^. It has been speculated that the possible causes of neurodegeneration caused by EV-A71 infection are a consequence of the virus-host interplays^14^. Enteroviral 2B proteins have been reported for viroporin activity to induce calcium ion (Ca^2+^) mobilization in host cells^15,16^. It has been known that Ca^2+^ is essential as being a secondary messenger involving in a signaling system to operate and regulate cellular functions. It is controlled by a complex interaction of signaling toolkits to optimize their functional properties^17,18^. Maintaining the Ca^2+^ homeostasis is a critical process in terms of keeping the normal cellular conditions and functions, especially in the CNS. In neuronal cells, the calcium signaling regulates many aspects of the neuronal functions such as membrane potential, neurotransmitter releasing, energy generation, gene transcription, and synaptic plasticity^19^. Imbalanced Ca^2+^ level can introduce dysregulation of the neuronal cell functions and has been implicated in several neuronal diseases, particularly in neurodegenerative disorders^20,21^. Our previous study revealed that human neuroblastoma SH-SY5Y cells transfected with EV-A71 2B protein showed a transient increase of cytosolic Ca^2+^ and underwent apoptotic cell death^10^. However, the inclusive mechanisms have not been elucidated.

Our transcriptomic data demonstrated the signature of the transcriptional response of human neuroblastoma SH-SY5Y cells transfected with 2BmCherry, by pairwise comparisons of the DEGs with those of mCherry and normal cells (SHSY5Y). The signature genes of the 2BmCherry included 333 upregulated and 333 downregulated DEGs in comparison to the SHSY5Y and 7 upregulated DEGs in comparison to the mCherry. The set of 7 upregulated DEGs from 2BmCherry versus mCherry comparison comprised C–C motif chemokine ligand 2 (*CCL2*), RELB proto-oncogene NF-κB subunit (*RELB*), interleukine-32 (*IL32*), tissue-type plasminogen activator (*PLAT*), prostaglandin E synthase (*PTGES*), pleckstrin homology-like domain family A member 1 (*PHLDA1*), and tumor necrosis factor receptor superfamily member 9 (*TNFRSF9*). The fewer numbers of the upregulated DEGs in the 2BmCherry transcriptome compared with mCherry might be due to the lower SH-SY5Y cell transfection efficiency of the 2BmCherry than mCherry as reported previously^10^. Hence, the levels of the expressed transcripts in the 2BmCherry transcriptome were relatively lower than those in the mCherry transcriptome. It is possible that some low-abundant transcripts might not be detected from the 2BmCherry transcriptome. These limitations warrant further studies to expand and complete the 2BmCherry transcriptome by employing other methods such as flow cytometry or single-cell isolation to sort the transfected cells followed by RNA-sequencing. Nonetheless, in this study, the upregulation of the representative 7 DEGs which had been identified by RNA-sequencing were subsequently validated by qRT-PCR. The complied results between RNA-sequencing and qRT-PCR indicated that the expression of the DEGs identified in this study were genuine.

The cellular processes and the pathways of the signature genes of the 2BmCherry were further identified by GO, KEGG, and protein–protein interaction network analysis. It was shown that the uniquely upregulated DEGs were enriched in the Ca^2+^-related signaling pathways including long-term potentiation (adaptation of neuronal synaptic activity), Wnt, MAPK, and non-canonical NIK/NF-κB pathway. Several pieces of evidence demonstrated that the changes of protein phosphatase activity had the roles in neuronal cell death and neuroinflammation associated with Alzheimer’s disease pathogenesis^22–25^. The downstream process of the activation of the Ca^2+^-related signaling pathways was likely to lead the gene expressions as the upregulated DEGs involved in calcineurin, canonical NF-κB, and non-canonical NIK/NF-κB pathways, and spliceosomal complex. The activation of calcineurin potentially leads to triggering the NFAT transcription factor. Consistently, the role of Ca^2+^ in regulation of the gene expression in the neurons has been reported^18,26^. Further, the genes in the cellular processes related to apoptosis were upregulated. This finding suggested the validity of the RNA sequencing data of the 2BmCherry transcriptome and conformed to the previous study which demonstrated the increased cytosolic Ca^2+^ and induction of apoptosis in the SH-SY5Y cells transfected with EV-A71 2BmCherry^10^. Moreover, the study demonstrated the gene upregulation of caspase-9 (*CASP9*) and *CASP12* at 3- and 24 h.p.t, respectively, in the SH-SY5Y cells transfected with the 2BmCherry where the former gene was being activated during the increased cytosolic Ca^2+^. This suggested that the mobilization of cytosolic Ca^2+^ between ER and mitochondria triggered the caspase-dependent apoptosis pathway. Transient elevation of cytosolic Ca^2+^ can trigger not only the cellular stress responses but also induce mitochondrial Ca^2+^ uptake^27,28^. It has been implicated that mitochondria are the key organelle to buffer the excessive cytosolic Ca^2+^ and boost up ATP production to maintain cellular energy resources and cell functions^29^. It was found in our 2BmCherry transcriptome that the genes related with electron transport chain and energy consumption, oxidoreductase activity, and mitochondrial protein complex were upregulated. This indicated the role of mitochondria to compensate the cytosolic Ca^2+^ imbalance and generate house-power for supplying the needs of energy to maintain the normal cellular functions. A related study showed that the EV-A71 infection triggered the mitochondrial oxidative stress and the abnormal energy metabolism to maximize the virus propagation and compensate the cellular functional defects in glioblastoma line SF268^30^. However, the long-term handling of Ca^2+^ dysregulation caused the mitochondrial Ca^2+^ overload resulting in mitochondrial dysregulation, leading to activation of oxidative stress responses and cell death^28^. It could be postulated that the effects of Ca^2+^ dysregulation mediated by EV-A71 2B protein that triggers the oxidative stress responses might play an important role in neuropathogenesis. The loss of mitochondrial activity by the long-term Ca^2+^ dysregulation had also been implicated in a possible mechanism of neuropathogenesis of Parkinson’s disease and Alzheimer’s disease^31,32^. In vivo study in transgenic mice model that had a defect in Ca^2+^ regulation demonstrated the disruption of neuronal structure and functional networks^33^. Similarly, the signature DEGs in our 2BmCherry transcriptome showed downregulation of the genes related to actin and microtubule as well as synapse. The DEGs localized in microtubule organizing center, membrane of a neuron, dendrite, exon, and synapse. Since the maintenance of the neuronal cytoskeletal structure is critical and essential for the neuronal cell functions such as neuronal transport and morphology, the loss of neuronal cytoskeleton results in the disturbance of protein trafficking, loss of neuronal network, and cell death^34–36^. Mitogen-activated protein kinase (MAPK) signaling pathway was found to be significant in the 2BmCherry transcriptome. MAPK signaling pathway participates in the calcium signaling cascade which plays a pivotal role in the regulation of neuronal plasticity, morphology, and survival^37,38^. Hyperactivation of the Ca^2+^-dependent MAPK pathway results in cellular stress response leading to cell death^39^. Hence, based on our transcriptomic data, it could be postulated that Ca^2+^ dysregulation in the human neuronal SH-SY5Y cells mediated by EV-A71 2B may trigger activation of MAPK pathway resulting in cellular stress and leading to cell death. Surprisingly, the upregulated genes that were uniquely found in the 2BmCherry transcriptome were involved in the Wnt signaling pathway and long-term potentiation (adaptation of neuronal synaptic activity) which was associated with cell survival of neurons and neurogenesis^40,41^. Wnt signaling pathway could be triggered in response to elevation of the cytosolic Ca^2+^ to maintain neuronal cell survival as a compensatory mechanism to restore the cellular homeostasis^42^. It seems likely that compensation of the dysfunctional neurons resulted from the induction of mobilizing cytosolic Ca^2+^ with anti-apoptotic effect. On the other hand, the compensation might be beneficial for the replication of EV-A71 at the early phase of the infection^43^.

**Figure 7:**
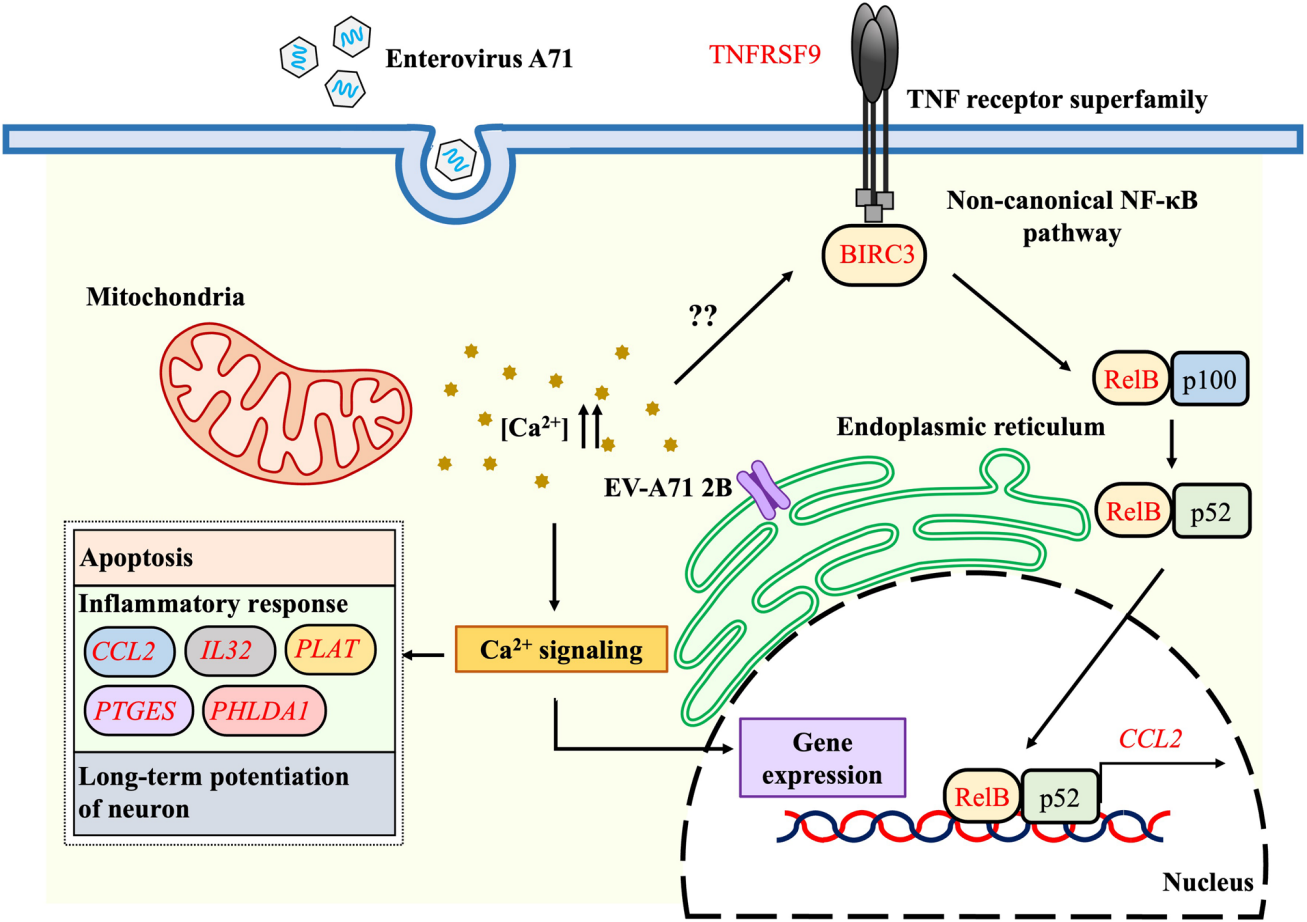
Summary of major upregulated genes and biological pathways in human neuroblastoma SH-SY5Y cells in response to 2B protein of enterovirus-A71 (EV-A71). The EV-A71 2B protein increases cytosolic calcium (Ca^2+^). The aberrant level of cytosolic Ca^2+^ triggers Ca^2+^-related signaling pathways participating gene expression, apoptosis, inflammatory response, non-canonical NF-κB signal transduction, and long-term potentiation of neuron. *CCL2*, *IL32, PLAT, PTGES,* and *PHLDA1* are associated with neuroinflammation. The EV-A71 2B might activate non-canonical NF-κB signaling pathway through TNFRSF9, BIRC3, and RelB by unidentified mechanism which lead to expression of CCL2.

In a PPI network, hub proteins, nodes with a large degree, are crucial proteins since they might be corresponding to the disease-causing genes^44^. In the 2BmCherry transcriptome, it was found that the most significant hub proteins encoded by the unique DEGs including *CCL2*, *RELB*, *BIRC3*, and *TNFRSF9*. The *RELB* and *BIRC3* were the genes in the non-canonical NF-κB pathway. Additionally, *NFKBI* (encoded for p50 precursor), a monomer of the heterodimer of NF-κB in the canonical pathway, was found to be a moderately significant node. RelB (encoded by *RELB*) is one of the monomers in the heterodimer, RelB and p52, of non-canonical NF-κB^45^. It plays a role in the non-canonical NF-κB signaling cascade and potentially crosslinks to canonical signaling cascade in the regulation of the cellular immunity^46,47^. The detailed data on non-canonical NF-κB are still limited. However, it is increasingly clear that its activation is different between canonical and non-canonical NF-κB pathways. The activation of the non-canonical NF-κB pathway is targeted by a specific set of receptors, in contrast to the canonical NF-κB signaling pathway which responses to the signals elicited by the diverse receptors. Currently, the well-characterized non-canonical NF-κB receptors are a subset of TNFR superfamily members^48^. In our study, the *TNFRSF9* was upregulated in the 2BmCherry transcriptome and had the protein–protein interaction with the non-canonical NF-κB. It was suggested that the activation of the non-canonical NF-κB might be integrated with TNFRSF9. Pieces of evidence showed that the upregulation of *TNFRSF9* was related to infiltration of inflammatory cells^49,50^. The evidence in the mouse embryonic cerebellum C17.2 neural stem cells showed that the increase of *TNFRSF9* expression resulted in apoptosis through detachment-induced mechanism^51^. CCL2 is known as the potential chemotaxis of inflammatory myeloid cells to mediate the local inflammation. Recent evidence demonstrated a decreased expression of *CCL2* related to downregulation of *RELB* in non-canonical NF-κB signaling pathway. Therefore, RelB may participate in the regulation of the inflammation through non-canonical NF-κB signal transduction and control of *CCL2* expression^52,53^. Upregulations of *CCL2* and *TNFRSF9* were also found in transcriptome of SK-N-SH cells infected with EV-A71^54^. Importantly, the upregulation of *CCL2* was associated with the severity of encephalitis in EV-A71-infected patients^55^. The non-canonical NF‑κB pathway has been reported as a target of RNA viruses^56^. Moreover, *IL32, PLAT, PTGES,* and *PHLDA1* have been reported to be associated with neuroinflammation. IL32 has become a more attractive molecule as involved in neuroinflammation^57^. A piece of evidence showed that IL32 triggered the production of proinflammatory cytokines such as TNFα, IL-1β, IP-10, and IL18 by downstream activation of NF-κB cascade through PCK, MAPK, and STAT signal transductions. The study in rat primary astrocytes showed that the IL32 mediated upregulation of nitric oxide (NO) production and downstream activation of inflammatory responses by triggering the MAPK signaling pathways^58^. Expression of cyclooxygenase 2 (COX-2) in the brain has been associated with neurodegeneration^59^. The role of PHLDA1 in the regulation of COX-2 expression was demonstrated in microglial cells^60^. Brain tissues of *COX-2* knockout mice showed downregulation of prostaglandin E (PGE) synthesis, which catalyzed by prostaglandin E synthase (PTGES), resulting in a decrease in neuronal damage^61,62^. It could be postulated that COX-2 might be an upstream regulator of PTGES, which drives the expression of PGE. PLAT (tissue-type plasminogen activator) has been implicated in neuroinflammation by inducing the blood–brain barrier leakage and migration of inflammatory cells in the brain tissue^63,64^. The evidence in macrophage showed that activation of the PLAT was triggered by annexin-A2 in a Ca^2+^-dependent manner^65^. It indicated that EV-A71 2B protein might play a role in immunopathogenesis of the CNS which probably associated with the non-canonical NF-κB pathway. The findings were summarized in the Fig. 7. The data suggest that transcriptomic profiling can provide a novel information source for studying the neuropathogenesis of EV-A71 infection leading to the development of the therapeutic measures for CNS complications.

## Methods

### Virus and cells

Human neuroblastoma SH-SY5Y cells (ATCC®, CRL-2266™) and human rhabdomyosarcoma (RD) cells (JCRB9072) were cultured in Dulbecco’s modified Eagle’s medium/F12; DMEM/F12, (Gibco, NY, USA) and DMEM (Biochrom, Cambridge, UK), respectively. Both media were supplemented with 10% fetal bovine serum, L-glutamine, and penicillin/streptomycin antibiotics (Gibco, NY, USA). The cells were incubated at 37 °C in the humidified air containing 5% CO2. Enterovirus A71 (EV-A71) subgenotype B5 was propagated in RD cells. The virus titer was determined by median cell culture infectious dose (CCID_50_) as described previously^10^.

### Transfection and RNA Isolation

The human SH-SY5Y neuroblastoma cells were seeded at a density of 5 × 10^5^ cells/well in 6-well plates and grown until reached approximately 50% to 70% confluence. The cell monolayers were transfected with *pLVX-puro::FLAG-2B-mCherry* by Lipofectamine™ 3000 reagents according to the manufacturer’s instructions (ThermoFisher Scientific, CA, USA) as described previously^10^. The *pLVX-puro::FLAG-2B-mCherry*, designated as 2BmCherry, was the recombinant plasmid expressing the EV-A71 2B protein fused with FLAG-epitope tag and mCherry fluorescent protein at N- and C-termini, respectively. The sequence of *2B* was amplified from the Thai clinical isolate of EV-A71 genotype B5, which was kindly provided by Prof.Yong Poovorawan, M.D. (Faculty of Medicine, Chulalongkorn University, Bangkok, Thailand) as described previously^10^. The cell monolayers transfected with *pLV-mCherry*, designated as mCherry (Addgene plasmid #36084; http://n2t.net/addgene:36084; RRID: Addgene_36084), was a plasmid expressing mCherry protein and served as the background control. Untransfected cell monolayers were the normal control. Each experimental condition was performed in three biological replicates. At 6 h post-transfection, total RNA was extracted from the cell monolayers using RNeasy® mini kit (QIAGEN, Hilden, Germany). RNA concentrations and purity were determined by measuring the OD at A260, A280, and A230 using NanoPhotometer® spectrophotometer (IMPLEN, CA, USA). The ratios of OD A260/280 and A260/A230 more than 1.8 were accounted as less contamination by proteins and salts, respectively. RNA integrity and genomic DNA contaminations were detected by agarose gel electrophoresis and Bioanalyzer RNA 6000 Nano assay (Agilent technologies, CA, USA). The transfections were verified by reverse-transcription PCR (RT-PCR) by using EV-A71 2B- or mCherrry sequence-specific primers^10^ and visualized by agarose gel electrophoresis. The RNA samples were stored at − 80 °C until use.

### RNA sequencing and quality control of data

The RNA samples extracted from three biological replicates of individual experimental groups including 2BmCherry-, mCherry transfected SH-SY5Ycells and untransfected cells were transferred into Active Chemical Protection™ tube for RNA (GenTegra LLC, Pleasanton, CA, USA) and transported for RNA sequencing by Novogene. The paired-end cDNA libraries were prepared using NEBNext® Ultra™ RNA library prep kit for Illumina® (New England BioLabs; NEB, MA, USA). Briefly, the poly(A) + mRNAs were enriched using oligo-d(T)25 NEBNext® poly(A) mRNA magnetic beads. The poly(A) + mRNAs were randomly fragmented and reverse transcribed into double-stranded cDNA (dscDNA) fragments. Thereafter, NEBNext® adaptors for Illumina® with the hair pin loop structure were ligated to the blunt ends of the dscDNA. Lastly, the dscDNA libraries were enriched by PCR using Phusion high-fidelity DNA polymerase and purified using Agencourt® AMPure® XP beads. The purified pair-end cDNA libraries were fed into high-throughput Illumina® HiSeq™ PE150 platform and sequenced to generate the sequencing data. The raw sequencing reads were processed for quality control by in-house Perl script QC. The parameters of quality control relied on per-base quality Phred Q score 30 (Q30), sequencing error rate distribution, and per read GC-content. Reads with low quality and containing unknown bases as well as adaptor related sequences were removed to get clean reads for downstream bioinformatics analysis. Finally, only high-quality reads were filtered in the dataset for further analysis.

### Bioinformatic analysis of transcriptomic data

#### Transcriptome assembly

All high-quality paired-end reads from three biological replicates of the individual experimental groups were mapped with the human reference genome GRCh38 using TopHat2 v2.0.12/Bowtie v2.2.3. The contigs of uniquely mapped reads were assembled into the complete transcript sequences of which were reconstructed into the final transcriptome using Cufflinks v2.2.1. The transcript sequences of each transcriptome were then annotated to a known human gene using NCBI BLAST v2.2.28 with an expected value (e-value) of 1.0 × 10^–10^.

#### Analysis of differentially expressed genes

Counts of the uniquely mapped reads on each transcript were normalized by fragment per kilobases per million mapped reads (FPKM) approach to estimate the abundance of the gene expression level. Threshold for the expressed genes were 0.1 FPKM-normalized expression values. The FPKM-normalized expression values were then normalized by transforming into log10(FPKM + 1) values, in which they were used in the gene expression analysis. Homogeneity of the transcripts from all biological replicates, both within and between the transcriptomes, was determined by kernel density estimated (KDE) curve and square of Pearson’s correlation coefficient value (*R*^*2*^) prior to comparison of the differentially expressed genes (DEGs). DESeq2 R package v1.18.1 was used to identify the DEGs by expressing a statistical value in magnitude of change and the log2(fold change). DEGs with upregulation were taken to greater than 0.1 log2(fold change) while those with downregulation were set to less than -0.1 log2(fold change). Statistical significance of DEGs was indicated by *p* values that were adjusted by the Benjamini and Hochberg approach. The adjusted *p* values less than 0.05 (*p*-adj < 0.05) were taken to indicate statistically significant difference. The DEGs analyses were three pairwise comparison groups including transcriptomes of 2BmCherry-transfected SH-SY5Y cells compared with mCherry-transfected cells and untransfected cells as well as mCherry-transfected cells and untransfected cells. Patterns of DEGs expressions among the three transcriptomes were demonstrated by unsupervised hierarchical heatmap clustering (h-cluster) analysis and Venn diagram using BioinfoGP Venny v2.1.0^66^.

#### Annotation of GO terms and analysis of KEGG signaling pathways

The uniquely expressed DEGs in the 2BmCherry transcriptome were categorized into biological process, molecular function, and cellular components which were annotated by Gene Ontology (GO). Biological pathways of the unique DEGs were identified by Kyoto encyclopedia of gene and genome (KEGG) database. Both GO and KEGG were analyzed using web-bases gene set enrichment analysis (GSEA) in molecular signature databases (MSigDB) v7.2 (www.gsea-msigdb.org/gsea/msigdb/). The FDR-corrected *p*-value less than 0.05 was taken to indicate the significance of GO term enrichment and KEGG pathway correlation.

### Protein–protein interaction network

Protein–protein interaction (PPI) network was conducted using the search tool for retrieval of interacting genes/protein (STRING) database to elucidate the potential mechanisms associated with the DEGs. The network of interactions was visualized using Cytoscape v3.8.0 software.

### Qualitative real-time RT-PCT

A set of DEGs which uniquely expressed in EV-A71 2B transcriptome was selected for validation of the gene expression in the transfected cells by qualitative real-time reverse-transcription PCR (qRT-PCR) using the specific primers. RNA samples were collected from the SH-SY5Y cell monolayers transfected with 2BmCherry at 6 h post-transfection. Control groups were normal SH-SY5Y and mCherry-transfected cells. The DNaseI-treated RNA samples were used as the templates to amplify the selected genes by one-step qRT-PCR using Brilliant II SYBR® Green qRT-PCR one-step kit (Stratagene, CA, USA). The conditions applied for one-step qRT-PCR were reverse transcription at 42 °C for 60 min, followed by real-time PCR with the initial denaturation at 95 °C for 10 min, 40 cycles of 95 °C for 30 s, 58 °C (for *PLAT* and *TNFRSF9*) or 61 °C (for *CCL2, RELB, IL32, PTGES,* and *PHLDA1*) for 1 min, and 72 °C for 30 s, followed by melting curve analysis. The relative fold changes of the expression levels of the selected genes were quantified by 2^−ΔΔCT^ method^67^. Changes in the mRNA expression levels were calculated after normalization to the internal control, human *GAPDH* house-keeping gene. The ΔCT of *2BmCherry*- and *mCherry*-transfected SH-SY5Y cells were subtracted with those of the SHSY5Y normal cells. The fold-changes (2^−ΔΔCT^) of the gene expression were derived from the subtractive ΔCT of *2BmCherry*-transfected cells relative to those of *mCherry*-transfected cells (ΔΔCT). The data were derived from technical triplicates of qRT-PCR from three independent experiments and expressed in mean ± SD. List of the specific primers used for qRT-PCR was provided as Supplementary Table 6.

## Supplementary Information

Supplementary Figure 1.

Supplementary Table 1.

Supplementary Table 2.

Supplementary Table 3.

Supplementary Table 4.

Supplementary Table 5.

Supplementary Table 6.

## Data Availability

Data generated or analyzed in this study are included in this published article both in text and Supplementary Information files as mentioned in the main text. The RNA sequencing data generated in this study is deposited in the NCBI GEO repository under the accession number GSE191270 (https://www.ncbi.nlm.nih.gov/geo/query/acc.cgi?acc=GSE191270).
