# Supplementary material for: Transcriptome of human neuroblastoma SH-SY5Y cells in response to 2B protein of enterovirus-A71
Source: Sci Rep. 2022 Feb 2;12:1765. doi: 10.1038/s41598-022-05904-6 (PMC8810792; doi:10.1038/s41598-022-05904-6)
Supplement: Supplementary file 1 — Supplementary Figure 1. [file 41598_2022_5904_MOESM1_ESM.pdf]

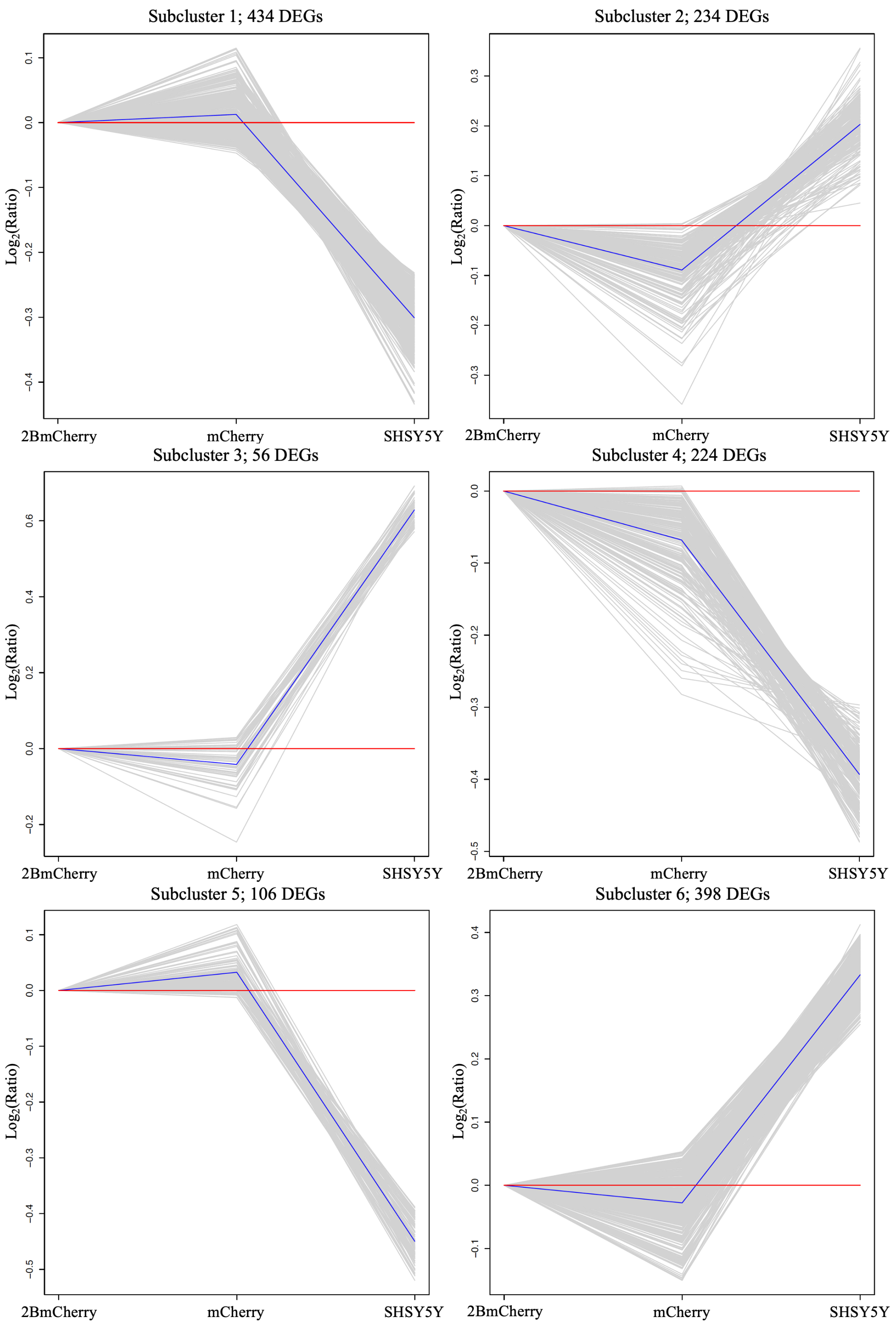
Subcluster 1; 434 DEGs
Subcluster 2; 234 DEGs
Subcluster 3; 56 DEGs
Subcluster 4; 224 DEGs
Subcluster 5; 106 DEGs
Subcluster 6; 398 DEGs
Log2(Ratio)
2BmCherry
mCherry
SHSY5Y
0.1
0.0
−0.1
−0.2
−0.3
−0.4
−0.5
0.2
0.3
0.4
0.6

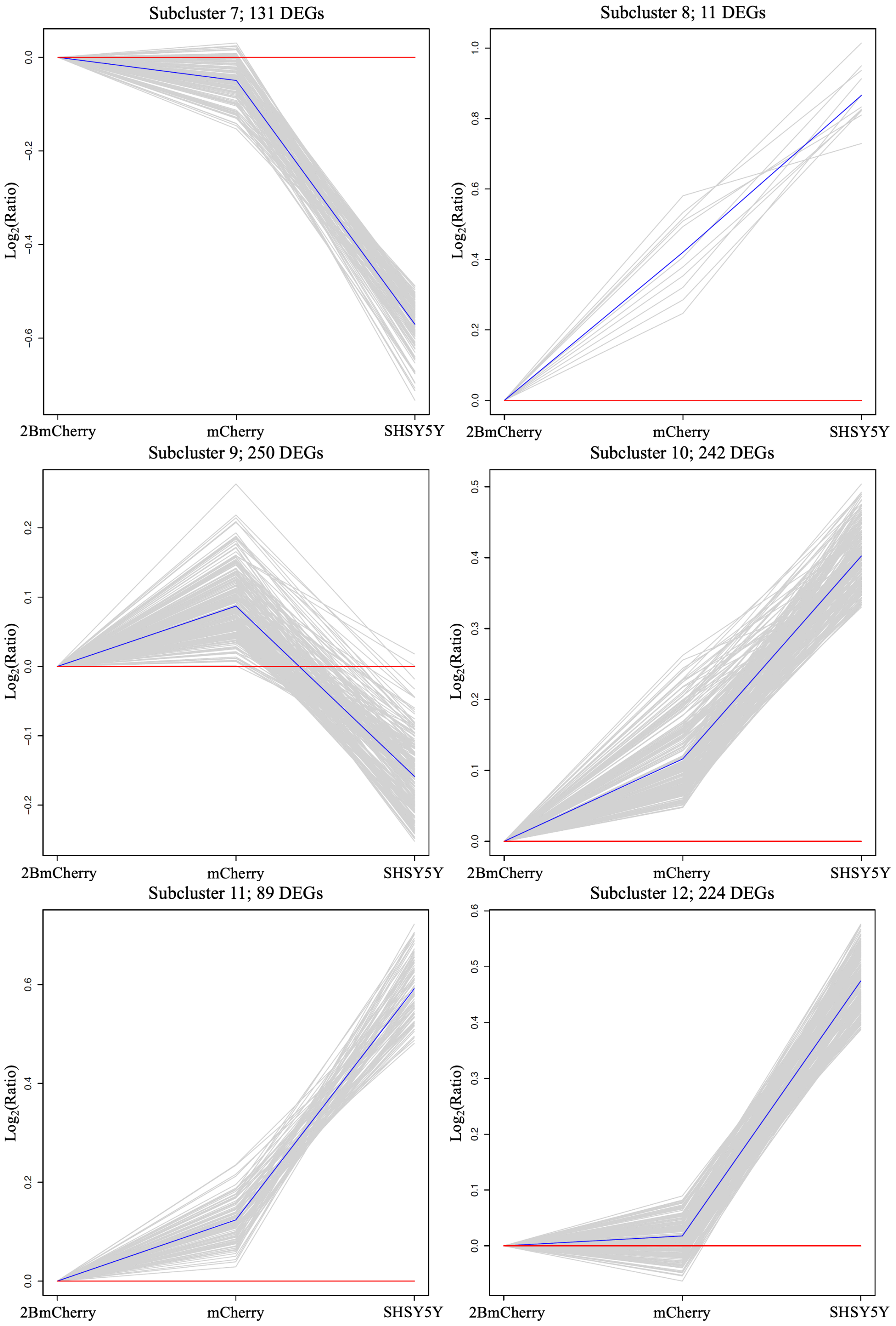
Subcluster 7; 131 DEGs
Subcluster 8; 11 DEGs
Subcluster 9; 250 DEGs
Subcluster 10; 242 DEGs
Subcluster 11; 89 DEGs
Subcluster 12; 224 DEGs
Log2(Ratio)
2BmCherry
mCherry
SHSY5Y

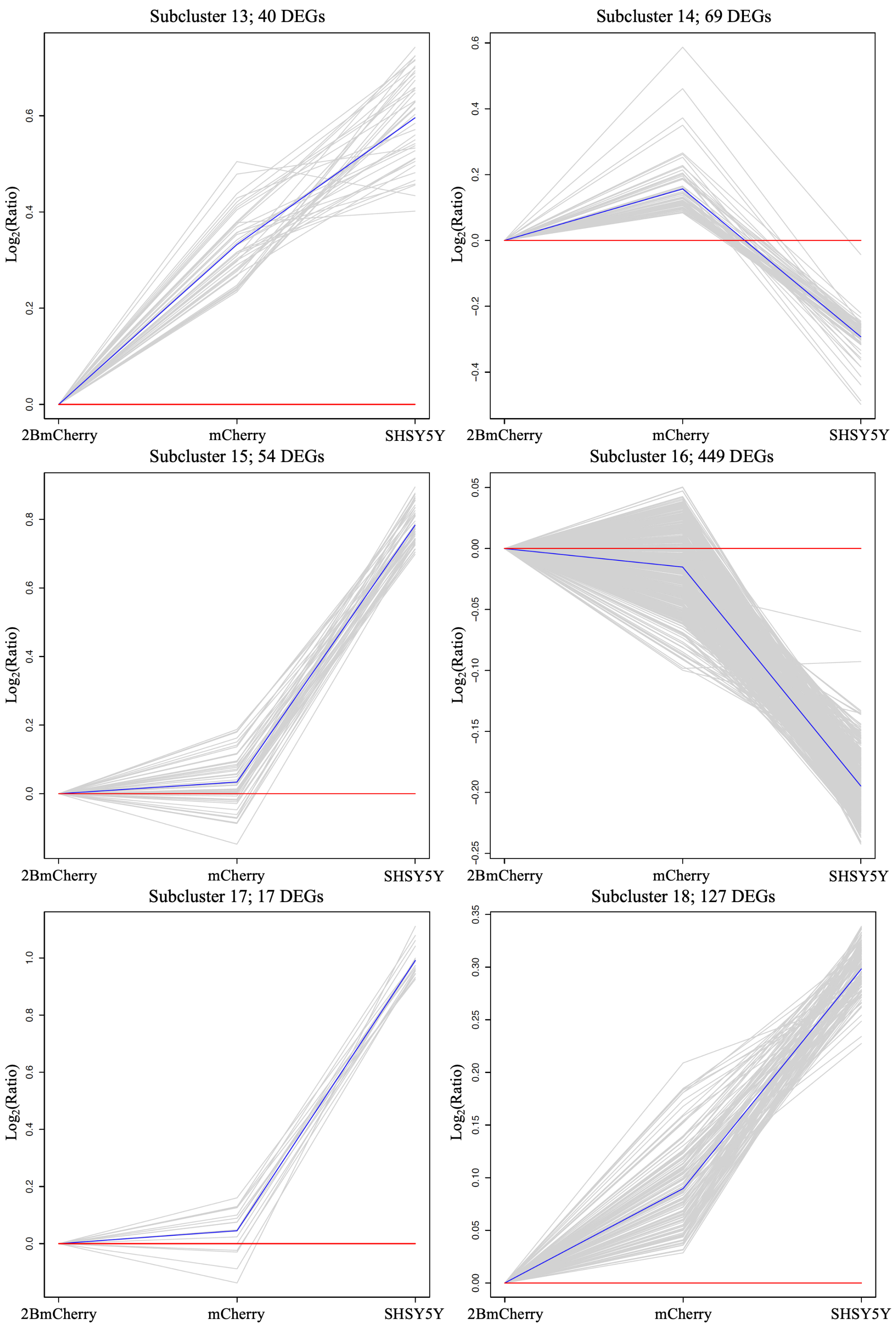

Subcluster 13; 40 DEGs
Subcluster 14; 69 DEGs
Subcluster 15; 54 DEGs
Subcluster 16; 449 DEGs
Subcluster 17; 17 DEGs
Subcluster 18; 127 DEGs
Log_2(Ratio)
2BmCherry
mCherry
SHSY5Y

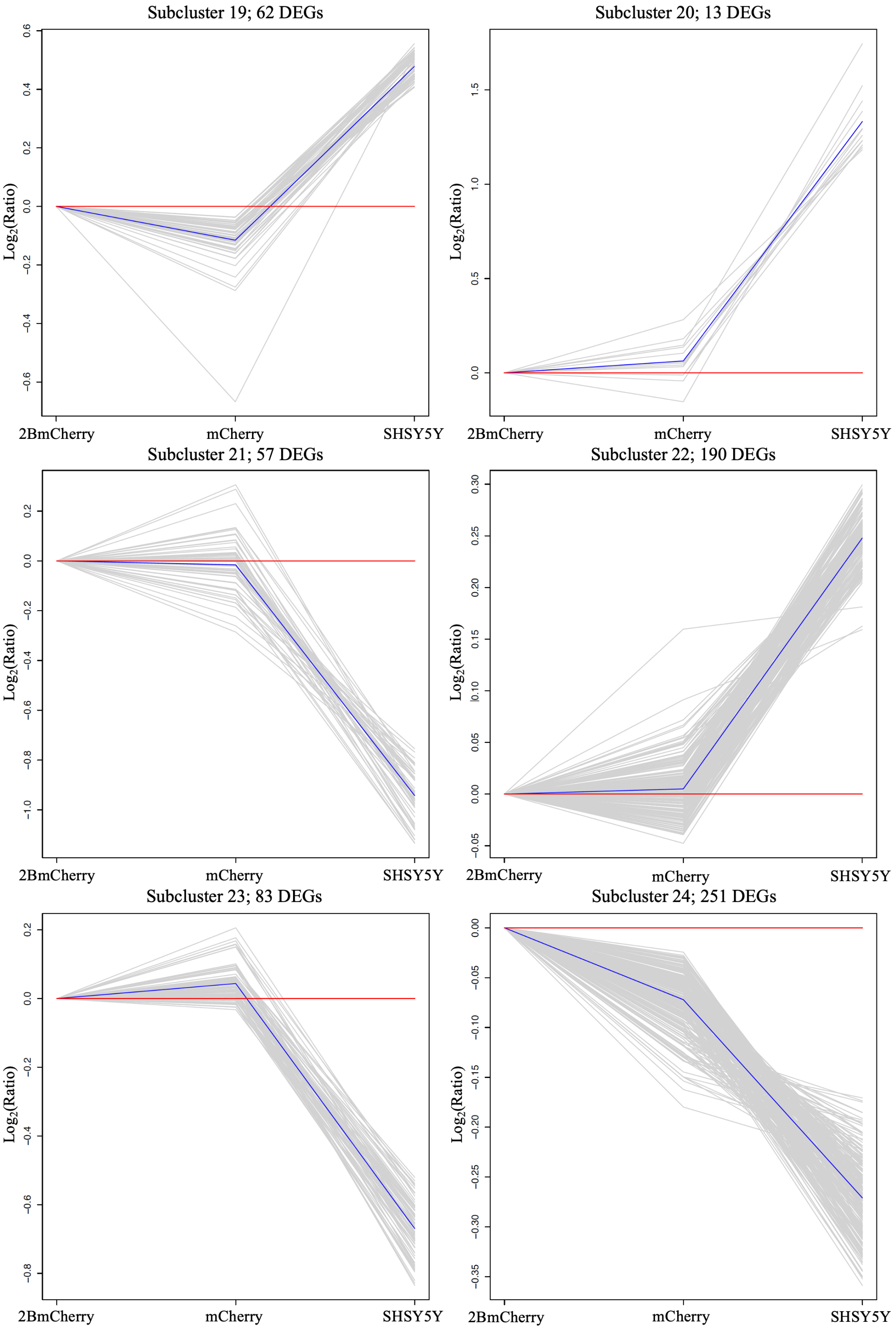
Subcluster 19; 62 DEGs
Subcluster 20; 13 DEGs
Subcluster 21; 57 DEGs
Subcluster 22; 190 DEGs
Subcluster 23; 83 DEGs
Subcluster 24; 251 DEGs
Log2(Ratio)
2BmCherry
mCherry
SHSY5Y

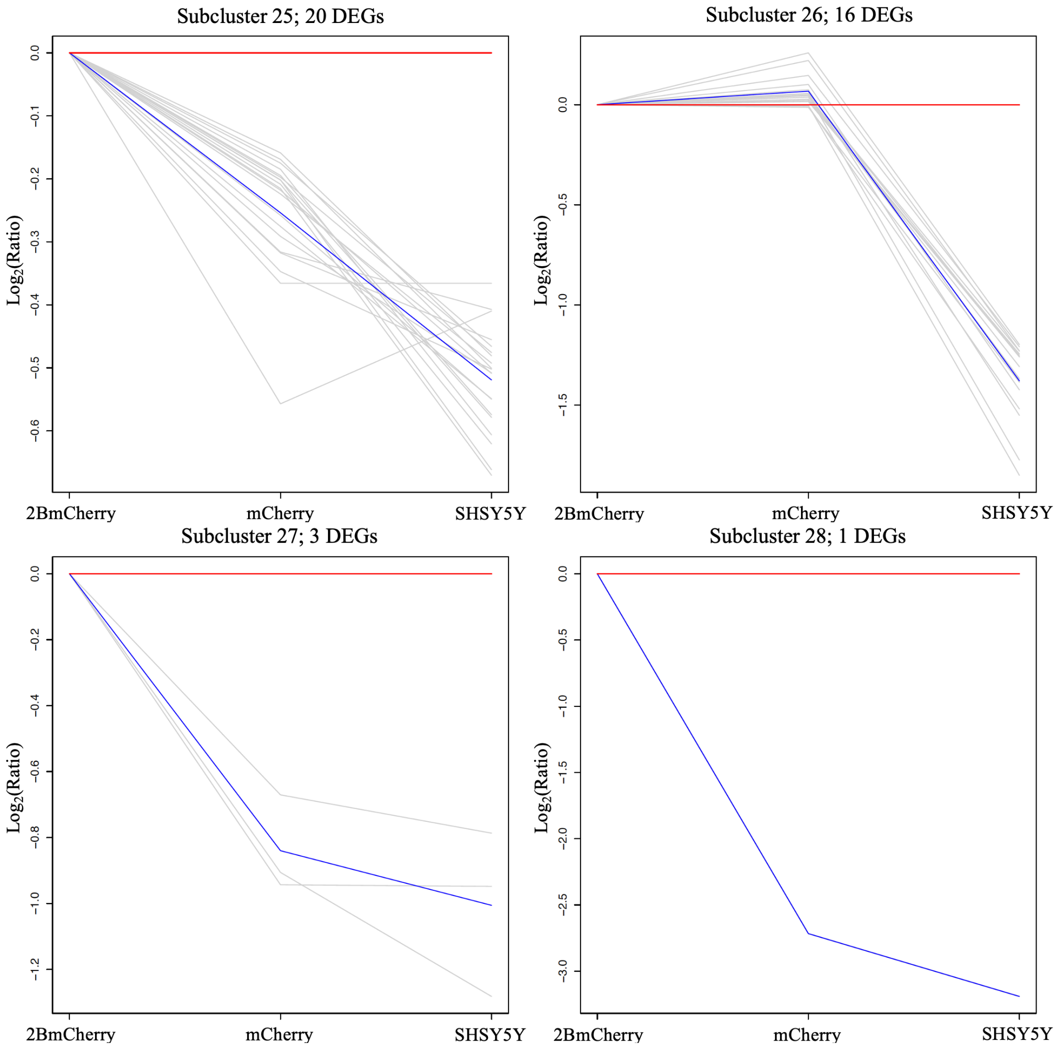

Supplementary Fig. 1. H-subcluster plots illustrated the 28 subclusters of all significant DEGs. Each gray line indicates the relative expression values of each significant DEG among three transcriptomes. The blue line indicates an average of the magnitude of changes in a cluster. The red line indicates a basal expression value among the transcriptomes. The *x*-axis represented the transcriptome. The *y*-axis showed the ratio of a relative expression value (log_2-transformed value) relative to those of the 2BmCherry transcriptome.
