# Supplementary material for: Transcriptome of human neuroblastoma SH-SY5Y cells in response to 2B protein of enterovirus-A71
Source: Sci Rep. 2022 Feb 2;12:1765. doi: 10.1038/s41598-022-05904-6 (PMC8810792; doi:10.1038/s41598-022-05904-6)
Supplement: Supplementary file 2 — Supplementary Table 1. [file 41598_2022_5904_MOESM2_ESM.pdf]

# Transcriptome of human neuroblastoma SH-SY5Y cells in response to 2B protein of enterovirus-A71

**Kittisak Suanpan[1], Potjanee Srimanote[1,2], Pongsri Tongtawe[1], Onruedee Khantisitthiporn[2,3], Oratai Supasorn[1], Patthaya Rattanakomol[1] & Jeeraphong Thanongsaksrikul[1,2*]**

[1]Graduate Program in Biomedical Sciences, Faculty of Allied Health Sciences, Thammasat University, Pathum Thani, 12120, Thailand

[2]Thammasat University Research Unit in Molecular Pathogenesis and Immunology of Infectious Diseases, Thammasat University, Pathum Thani, 12120, Thailand

[3]Department of Medical Technology, Faculty of Allied Health Sciences, Thammasat University, Pathum Thani, 12120, Thailand

*** Correspondence:** Jeeraphong Thanongsaksrikul
jeeraphong.t@allied.tu.ac.th

**Supplementary Table 1. The summary of read quality of RNA sequencing data from triplicate samples of each treatment and control groups after quality control process.**

| | Sample | Raw reads | Clean reads (%) | %Error rate | %Q30 | %GC content |
|---|---|---|---|---|---|---|
| **2BmCherry** | **2BmCherry1** | $41.31\times10^6$ | $40.20\times10^6$ (97.31) | 0.03 | 90.87 | 51.06 |
| | **2BmCherry2** | $46.76\times10^6$ | $45.37\times10^6$ (97.02) | 0.03 | 92.20 | 51.47 |
| | **2BmCherry3** | $44.53\times10^6$ | $43.15\times10^6$ (96.90) | 0.03 | 91.91 | 50.63 |
| **mCherry** | **mCherry1** | $46.22\times10^6$ | $44.87\times10^6$ (97.09) | 0.03 | 90.98 | 51.12 |
| | **mCherry2** | $40.69\times10^6$ | $39.60\times10^6$ (97.31) | 0.03 | 90.23 | 50.83 |
| | **mCherry3** | $45.46\times10^6$ | $44.39\times10^6$ (97.64) | 0.03 | 90.46 | 50.84 |
| **SHSY5Y** | **SHSY5Y1** | $41.29\times10^6$ | $40.38\times10^6$ (97.79) | 0.03 | 90.42 | 50.80 |
| | **SHSY5Y2** | $46.75\times10^6$ | $45.61\times10^6$ (97.56) | 0.03 | 92.17 | 50.81 |
| | **SHSY5Y3** | $46.77\times10^6$ | $45.56\times10^6$ (97.42) | 0.03 | 91.24 | 50.80 |

2BmCherry: *pLenti*::*FLAG-2B-mCherry* transfected SH-SY5Y cells

mCherry: *pLenti-mCherry* transfected SH-SY5Y cells

SHSY5Y: Untransfected SH-SY5Y cells
