# Supplementary material for: Transcriptome of human neuroblastoma SH-SY5Y cells in response to 2B protein of enterovirus-A71
Source: Sci Rep. 2022 Feb 2;12:1765. doi: 10.1038/s41598-022-05904-6 (PMC8810792; doi:10.1038/s41598-022-05904-6)
Supplement: Supplementary file 3 — Supplementary Table 2. [file 41598_2022_5904_MOESM3_ESM.pdf]

Transcriptome of human neuroblastoma SH-SY5Y cells in response to 2B protein of enterovirus-A71

Kittisak Suanpan[1], Potjanee Srimanote[1,2], Pongsri Tongtawe[1], Onruedee Khantisitthiporn[2,3], Oratai Supasorn[1], Patthaya Rattanakomol[1] & Jeeraphong Thanongsaksrikul[1,2*]

[1]Graduate Program in Biomedical Sciences, Faculty of Allied Health Sciences, Thammasat University, Pathum Thani, 12120, Thailand

[2]Thammasat University Research Unit in Molecular Pathogenesis and Immunology of Infectious Diseases, Thammasat University, Pathum Thani, 12120, Thailand

[3]Department of Medical Technology, Faculty of Allied Health Sciences, Thammasat University, Pathum Thani, 12120, Thailand

*** Correspondence:** Jeeraphong Thanongsaksrikul
jeeraphong.t@allied.tu.ac.th

Supplementary Table 2. The summary of read mapping of RNA sequencing data from triplicate samples of each treatment and control groups against human reference genome GRCh38.

Sample name	2BmCherry1	2BmCherry2	2BmCherry3	mCherry1	mCherry2	mCherry3	SHSY5Y1	SHSY5Y2	SHSY5Y3
**Total clean reads**	40.20×10^{6}	45.37×10^{6}	43.15×10^{6}	44.87×10^{6}	39.60×10^{6}	44.39×10^{6}	40.38×10^{6}	45.61×10^{6}	45.56×10^{6}
**Total mappable reads**	90.55%	92.11%	92.05%	90.60%	89.89%	90.07%	90.66%	92.20%	91.30%
**Multiple mappable reads**	6.34%	6.54%	6.40%	6.39%	6.31%	6.31%	6.30%	6.33%	6.34%
**Uniquely mappable reads**	84.20%	85.56%	85.65%	84.20%	83.58%	83.76%	84.36%	85.87%	84.96%
**Non-splice reads**	42.70%	42.66%	44.03%	42.69%	43.60%	43.27%	43.37%	44.86%	43.92%
**Splice reads**	41.50%	42.90%	41.62%	41.51%	39.97%	40.49%	40.99%	41.00%	41.03%
**Exonic region**	96.30%	96.30%	95.80%	96.10%	95.20%	95.40%	95.70%	94.90%	94.90%
**Intronic region**	2.40%	2.40%	2.80%	2.50%	3.30%	3.10%	2.70%	3.40%	3.50%
**Intergenic region**	1.30%	1.30%	1.40%	1.40%	1.50%	1.50%	1.50%	1.70%	1.60%

Total read clean reads: The number of filtered high-quality clean reads.
Total mappable reads: The number of reads that can be mapped to the reference genome.
Multiple mappable reads: The number of reads that can be mapped to multiple site along the entire genome.
Uniquely mappable reads: The number of reads that mapped perfectly to single location on the reference genome..
Non-splice reads: The number of uniquely mapped reads that map entirely on one exon.
Splice reads: The number of uniquely mapped reads that can be segmented and mapped on two exons, called junction reads.
Exonic, intronic and intergenic region: The percentage of uniquely mapped reads that align on an exonic, intronic, and intergenic region, respectively.
