# Supplementary material for: Transcriptome of human neuroblastoma SH-SY5Y cells in response to 2B protein of enterovirus-A71
Source: Sci Rep. 2022 Feb 2;12:1765. doi: 10.1038/s41598-022-05904-6 (PMC8810792; doi:10.1038/s41598-022-05904-6)
Supplement: Supplementary file 7 — Supplementary Table 6. [file 41598_2022_5904_MOESM7_ESM.pdf]

Transcriptome of human neuroblastoma SH-SY5Y cells in response to 2B protein of enterovirus-A71

Kittisak Suanpan[1], Potjanee Srimanote[1,2], Pongsri Tongtawe[1], Onruedee Khantisitthiporn[2,3], Oratai Supasorn[1], Patthaya Rattanakomol[1] & Jeeraphong Thanongsaksrikul[1,2*]

[1]Graduate Program in Biomedical Sciences, Faculty of Allied Health Sciences, Thammasat University, Pathum Thani, 12120, Thailand

[2]Thammasat University Research Unit in Molecular Pathogenesis and Immunology of Infectious Diseases, Thammasat University, Pathum Thani, 12120, Thailand

[3]Department of Medical Technology, Faculty of Allied Health Sciences, Thammasat University, Pathum Thani, 12120, Thailand

*** Correspondence:** Jeeraphong Thanongsaksrikul
jeeraphong.t@allied.tu.ac.th

Supplementary Table 6. List of specific primers used for qRT-PCR in this study.

Gene		Sequences	Reference
Human *CCL2*	Forward<br>Reverse	5´-TCGCTCAGCCAGATGCAAT-3´<br>5´-CAATGGTCTTGAAGATCACAGC-3´	In this study
Human *RELB*	Forward<br>Reverse	5´-CGGCACAGTTTTAACAACCT-3´<br>5´-GATCCTCACCACATTCATGTC-3´	In this study
Human *IL32*	Forward<br>Reverse	5´-GAGCTCACTCCTCTACTTGAAA-3´<br>5´-CTTGTCACAAAAGCTCTCCC-3´	In this study
Human *PLAT*	Forward<br>Reverse	5´-TCAGCCTACCGTGGCA-3´<br>5´-CATCAGGATTCCGGCAGTA-3´	In this study
Human *PTGES*	Forward<br>Reverse	5´-CACGCTGCTGGTCATCAAGA-3´<br>5´-CAGAAAGGAGTAGACGAAGCC-3´	In this study
Human *PHLDA1*	Forward<br>Reverse	5´-AGGAAGGGCTGCTGCTTATC-3´<br>5´-CACACAGTCCACGGTCTTCA-3´	In this study
Human *TNFRSF9*	Forward<br>Reverse	5´-AAACGGGGCAGAAAGAAACT-3´<br>5´-CTTCTGGAAATCGGCAGCTA-3´	Fukamachi, T. *et al*. 2014*
Human *GAPDH*	Forward<br>Reverse	5´-CAAGGTCATCCATGACAACTTTG-3´<br>5´-GTCCACCACCCTGTTGCTGTA-3´	In this study

* Fukamachi, T., Ikeda, S., Saito, H., Tagawa, M., & Kobayashi, H. Expression of acidosis-dependent genes in human cancer nests. *Mol. Clin. Oncol*. **2**, 1160-1166 (2014).
